# Serum Soluble TACI, a BLyS Receptor, Is a Powerful Prognostic Marker of Outcome in Chronic Lymphocytic Leukemia

**DOI:** 10.1155/2014/159632

**Published:** 2014-08-06

**Authors:** Marie-Christine Kyrtsonis, Katerina Sarris, Efstathios Koulieris, Dimitrios Maltezas, Eftychia Nikolaou, Maria K. Angelopoulou, Vassiliki Bartzis, Tatiana Tzenou, Maria Dimou, Mariana P. Siakandaris, Nora A. Viniou, Sotirios Sachanas, Christina Kalpadakis, Petros P. Sfikakis, Gerassimos A. Pangalis, Panayiotis Panayiotidis

**Affiliations:** ^1^Hematology Section of the First Department of Propedeutic Internal Medicine, Laikon University Hospital, Agiou Thoma 17, 11527 Athens, Greece; ^2^Hematology Clinic of Athens' Medical Center, Andersen 1, Psychiko, 11525 Athens, Greece

## Abstract

BLyS is involved in CLL biology and its low soluble serum levels related to a shorter time to first treatment (TFT). TACI is a BLyS receptor and can be shed from cells' surface and circulate in soluble form (sTACI). We investigated the impact of serum BLyS and sTACI levels at diagnosis in CLL patients and their relationship with disease parameters and patients' outcome. Serum BLyS was determined in 73 patients, while sTACI in 60. Frozen sera drawn at diagnosis were tested by ELISA. sTACI concentrations correlated with BLyS (*P* = −0.000021), b2-microglobulin (*P* = 0.005), anemia (*P* = −0.03), thrombocytopenia (*P* = 0.04), Binet stage (*P* = 0.02), and free light chains ratio (*P* = 0.0003). Soluble BLyS levels below median and sTACI values above median were related to shorter TFT (*P* = 0.0003 and 0.007). During a ten-year followup, sTACI levels, but not BLyS, correlated with survival (*P* = 0.048). In conclusion, we confirmed the prognostic significance of soluble BLyS levels with regard to TFT in CLL patients, and, more importantly, we showed for the first time that sTACI is a powerful prognostic marker, related to parameters of disease activity and staging and, more importantly, to TFT and OS.

## 1. Introduction

Chronic lymphocytic leukemia (CLL) is an indolent leukemic B-cell lymphoproliferative disorder, defined in the WHO classification of neoplasms as a neoplasm of mature B-lymphocytes involving peripheral blood, bone marrow, spleen, and lymph nodes [1]. It is the most common form of leukemia in the elderly in the Western world. For diagnosis a minimum of 5 × 10^9^/L absolute blood lymphocyte count is required; these typically coexpress CD5, CD19, CD20, and CD23 antigens with dim CD20, CD79b, and surface immunoglobulin expression [2, 3]. Patients usually enjoy a relatively favourable outcome and the majority of them are asymptomatic and may not need any treatment for years. However, some patients have a more aggressive course and shorter survival; clinical manifestations, when present, include anaemia, peripheral lymphadenopathy, splenomegaly, and autoimmune manifestations. Treatment should be immediately started in presence of a lymphocyte doubling time of less than 6 months, very enlarged or rapidly growing lymph nodes or spleen, anemia, thrombocytopenia, and B-symptoms. For symptomatic patients, adverse prognostic factors include classical staging (according to Rai or Binet), unmutated* VH* genes, ZAP-70 and CD38 expression, and cytogenetic alterations such as deletion of 11q22, deletion of 17p, and/or presence of a* TP53* mutation [4]. Additional prognostic factors are needed for asymptomatic patients to predict whether they will remain stable for years or not.

B-lymphocyte stimulator (BLyS) is a cytokine, member of the TNF-superfamily, that is involved in CLL biology and was shown to regulate B-CLL cells proliferation and survival [5]. Furthermore, serum BLyS levels were found decreased in CLL patients and their low concentrations related to a shorter time to first treatment (TFT) but not to overall survival (OS) [6]. BLyS is produced by myeloid cells, monocytes, dendritic cells, and osteoclasts [7]. It may be cleaved from cells' surface and circulate in body fluids in a soluble form [8]. BLyS actions concern almost exclusively cells of lymphoid lineage and are exerted through its receptors [9]. TACI (transmembrane activator and CAML interactor) is one of the 3 BLyS (BAFF) receptors and is expressed by B and T cells. It can also bind APRIL. TACI can also be shed from cells' surface and circulate in its soluble form [10, 11]. Very few studies so far investigated soluble TACI (sTACI) serum levels in CLL.

The purpose of this study was to investigate possible relationship between serum BLyS and sTACI concentrations at diagnosis in CLL, as well as eventual correlations of their respective levels with disease parameters and patients' outcome.

## 2. Patients and Methods

Seventy-three CLL patients were studied. Their characteristics are shown in Table 1.

Sera from patients were drawn at diagnosis and aliquots were kept frozen and retrospectively tested to determine BLyS and sTACI concentrations. Frozen sera from 14 healthy individuals (HI) for BLyS and sTACI, respectively, were also tested as controls.

42 patients were or became symptomatic and in need of treatment during disease course. Patients' median OS was 79 months (range 18–174) while median TFT was 34 months (range 1–157).

Serum BLyS levels were determined by ELISA (R&D Quantikine KIT) in all 73 patients while sTACI (R&D Quantikine, DuoSet) in 60 of them, according to the manufacturer's instruction; briefly (1) for BLys determination, the specified amount of patients' or HI serum was added to a 96-cell microplate already coated with a capture antibody. After incubation the plate was washed, the detection antibody was added, and after another incubation and wash Streptavidin-HRP and Tetramethylbenzidine in H_2_O_2_ were added, and finally H_2_SO_4_ was added to stop the reaction. (2) For sTACI determination, a 96-cell microplate was coated with the specified amount of capture antibody and, after being incubated overnight and washed, Reagent Diluent was added to block the plate and after another wash the specified amount of patients' or controls' sera was added. The subsequent procedure was almost identical (washing, addition of detection antibody, incubation, washing, and addition of Streptavidin-HRP followed by Tetramethylbenzidine in H_2_O_2_ solution and then stop solution). In order to determine the values of both BLys and sTACI, the optical density of each cell was determined at 450 + 620 nm by using a photometer. On a biaxial system where *x*-axis was the serum concentration of the cytokine and *y* was optical density, the sera cytokine concentration was determined using a curve plotted based on the optical density of known serial concentrations.

Statistical analysis was performed using the SPSS v.15 software. Nonparametric variables were compared by the Mann-Whitney test. TFT and OS curves according to BLyS or sTACI levels were assessed and plotted by the Kaplan-Meier analysis and then compared by the log-rank test. *P* values of less than 0.05 were considered statistically significant.

## 3. Results and Discussion

### 3.1. Serum BLyS and sTACI Levels

**Figure 1:**
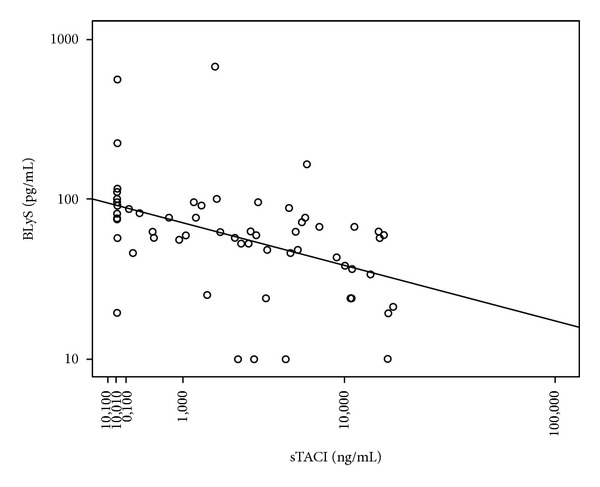
Inverse correlation between serum soluble BLyS and sTACI.

In the present study, median serum BLyS levels were 65 pg/mL (undetectable −680) while sTACI's ones were 2,52 ng/mL (undetectable −17) in CLL patients. The corresponding median concentrations in HI sera samples were 183 pg/mL (undetectable −381) and 0.86 ng/mL (undetectable −4,14) for BLyS and sTACI, respectively. The difference between levels in patients and HI is significant for both (*P* < 0.01). In addition, sTACI concentrations strongly correlated inversely with soluble BLyS (*P* = 0.000021), as shown in Figure 1.

In accordance with our results, serum BLyS levels were reported lower in CLL patients as compared to HI, in several previous studies [12–14]. However, with regard to sTACI levels in CLL, that we found higher in patients than in HI, there are no reports in medical literature, so far, to our knowledge.

As an attempt to explain the low serum BLyS levels found in CLL patients, Molica et al. [14] suggested that in aggressive CLL high amounts of soluble BLyS are bound by its receptors on B-cell surface, thus sequestrating it from circulation. This theory was reinforced by another study that showed that serum BLyS concentrations increased in follicular lymphoma patients after rituximab administration, possibly because it is unbounded by its surface receptors [15]. In view of our finding of a strong inverse correlation between BLyS and sTACI serum concentrations, one could assume that binding of soluble BLyS to sTACI results in hiding the BLyS epitope that is detected by ELISA measurements and that BLyS levels are falsely low. This supposition remains indeed to be proved but if it was the case, it would have significant therapeutical implications, given that anti-BLyS or anti-BLyS receptors antibodies have been manufactured for adjuvant treatment in B-cell lymphoproliferative disorders [16, 17] but are not considered for CLL for which caution is needed until BLyS contribution to disease biology is fully understood.

### 3.2. Correlations between BLyS or sTACI Levels and Disease Parameters

Serum BLyS levels correlated inversely with absolute lymphocyte count (*P* = 0.01, Spearman's rho = −0.303). Furthermore an inverse correlation was demonstrated for BLyS values above median and bone marrow lymphocyte infiltration greater than 50% (*P* = 0.031, chi square) while sTACI concentrations correlated with b2-microglobulin (*P* = 0.005), inversely with anemia (*P* = 0.03), thrombocytopenia (*P* = 0.04), Binet stage (*P* = 0.02), and free light chains ratio (*P* = 0.0003). In Molica et al.'s study higher BLyS levels were associated with younger age, higher platelet count, mutated IgVH, normal cytogenetic profile or presence of 13q deletion, and low ZAP-70 and CD38 expression. Unfortunately, in our series mutational status and cytogenetic abnormalities were determined in only 15% of patients rendering statistical analysis impossible; ZAP-70 expression was not determined and there was no correlation with CD38 expression. With regard to sTACI levels, given that this is the first report, we cannot compare with other studies.

### 3.3. Time to First Treatment and Overall Survival according to BLyS and sTACI Serum Concentrations

**Figure 2:**
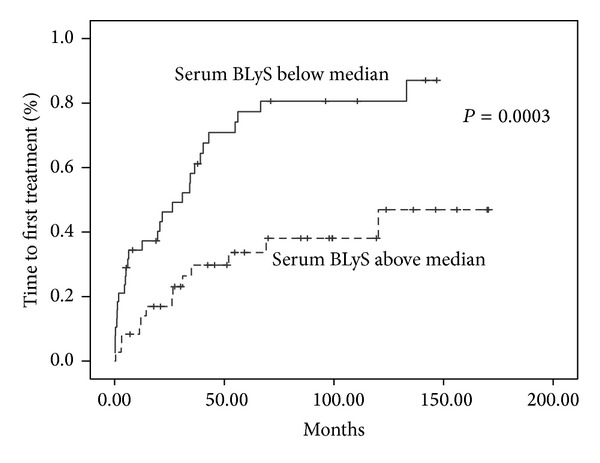
Time to first treatment according to serum soluble BLyS.

**Figure 3:**
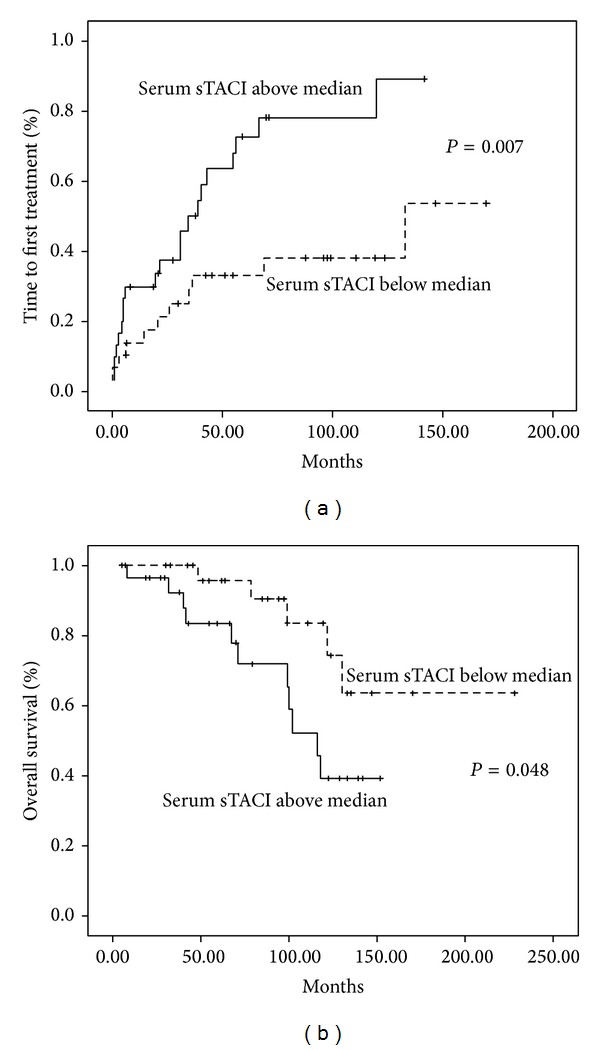
Time to first treatment (a) and overall survival (b) according to serum sTACI.

In CLL patients serum BLyS values below median were related to a significantly shorter TFT compared to values above median (*P* = 0.0003) (Figure 2) but no correlation was found with overall survival. In addition, serum sTACI values above median were also related to a shorter TFT compared to values below median (*P* = 0.007) and, importantly, also to OS (*P* = 0.048, HR: 2.789, 95% CI: 0.967–8.047), as shown in Figure 3. Serum sTACI values above median maintained their negative prognostic impact in the Cox Regression model when tested with known disease parameters such as Rai and Binet stadium, b2-microglobulin, and LDH.

Lower soluble BLyS levels have already been associated with a shorter time to first treatment [14, 18] but not with overall survival, most probably because of the indolent nature of the disease, requiring a very long follow-up time, in order to show survival advantage. Of special interest are our findings concerning the prognostic significance of sTACI with regard to both TFT and OS; such findings have not been reported so far in medical literature. Because the vast majority of our patients were not in need of treatment and low staged at diagnosis, our results suggest that serum sTACI could be a new, easily assessed, marker for predicting CLL behaviour in low staged patients. Such prognostic factors are missing for asymptomatic patients although in this context serum free light chain measurements appear very promising [19–21].

## 4. Conclusions

We confirmed that low soluble BLyS are associated with a shorter time to first treatment while in addition we found that sTACI serum concentrations at diagnosis constitute a powerful prognostic marker in chronic lymphocytic leukemia; sTACI is related to disease activity parameters and the stage of CLL and more importantly, sTACI levels above median predicted a shorter time to first treatment and worse outcome for the patients. Indeed these results are preliminary and concern a relatively short series, although with a very long follow-up, and further researches are needed. However, if confirmed, our results suggest that sTACI could be a valuable prognostic marker in CLL while, in addition, they could open interesting therapeutical applications.

## Figures and Tables

**Table 1:** Patients' characteristics at the time of diagnosis.

Age, median (range)	60 years (37–82)	

	For BLyS measurements	For sTACI measurements

*N*	73	60

	%	

Sex, M/F	61/39%	66/34%
Binet stage		
1	59%	60%
2	34%	32%
3	7%	8%
Lymphadenopathy	60%	60%
Splenomegaly	18%	15%
Haemoglobin <10 g/dL	5.5%	8.3%
Platelet counts <100 × 10^9^/L	2.7%	3.3%
Abnormal LDH	15%	15%
BM infiltration >50%	59%	50%
*β*2-Microglobulin >3.5 mg/L	30% (12/39 pts)	27% (9/33 pts)
Abnormal *κ*-sFLC (normal 3.3–19.4 mg/L)	30% (20/67 pts)	30%(17/56 pts)
Abnormal *λ*-sFLC (normal 5.71–26.3 mg/L)	8% (3/67 pts)	5% (3/56 pts)

FLC: free light chain.
